# Specific Ion Effects of Dodecyl Sulfate Surfactants with Alkali Ions at the Air–Water Interface

**DOI:** 10.3390/molecules24162911

**Published:** 2019-08-10

**Authors:** Eric Weißenborn, Björn Braunschweig

**Affiliations:** Institute of Physical Chemistry and Center for Soft Nanoscience, Westfälische Wilhelms-Universität Münster, Corrensstraße 28/30, 48149 Münster, Germany

**Keywords:** dodecyl sulfate, specific ion effects, sum frequency generation, Hofmeister series, surface excess, stark shift

## Abstract

The influence of Li^+^, Na^+^ and Cs^+^ cations on the surface excess and structure of dodecyl sulfate (DS^−^) anions at the air–water interface was investigated with the vibrational sum-frequency generation (SFG) and surface tensiometry. Particularly, we have addressed the change in amplitude and frequency of the symmetric S-O stretching vibrations as a function of electrolyte and DS^−^ concentration in the presence of Li^+^, Na^+^ and Cs^+^ cations. For the Li^+^ and Na^+^ ions, we show that the resonance frequency is shifted noticeably from 1055 cm^−1^ to 1063 cm^−1^ as a function of the surfactants’ surfaces excess, which we attribute to the vibrational Stark effect within the static electric field at the air–water interface. For Cs^+^ ions the resonance frequency is independent of the surfactant concentration with the S-O stretching band centered at 1063 cm^−1^. This frequency is identical to the frequency at the maximum surface excess when Li^+^ and Na^+^ ions are present and points to the ion pair formation between the sulfate headgroup and Cs^+^ counterions, which reduces the local electric field. In addition, SFG experiments of the O-H stretching bands of interfacial H_2_O molecules are used in order to calculate the apparent double layer potential and the degree of dissociation between the surfactant head group and the investigated cations. The latter was found to be 12.0%, 10.4% and 7.7% for lithium dodecyl sulfate (LiDS), sodium dodecyl sulfate (SDS) and cesium dodecyl sulfate (CsDS) surfactants, which is in agreement with Collins ‘rule of matching water affinities’.

## 1. Introduction

Specific ion effects are known since the early work of Hofmeister who has ordered salts according to their ability to salt out proteins [1,2]. Ion effects play an important role in many fields from surfactant [3,4,5,6,7,8], colloid [9] science to biology [2], where ion effects are relevant for enzyme activation and protein stabilization [2,10,11]. In colloid science, the presence of specific salts is for instance used in order to control the lower critical solution temperature (LCST) of thermo-responsive polymers [12,13,14,15]. So far there are many approaches that have gained insights into ion specific effects and their chemical nature from experiments [5,16,17,18] and theoretical modelling [2,15,19,20,21] as well as by proposing empirical concepts [22,23]. Collins [22,23] proposed a ‘rule of matching water affinities’ which describes the tendency of different oppositely charged ions to associate and to form contact or solvent-shared ion pairs in aqueous solutions. The basis of this rule is linked to the differences in the affinity of ions to bind water molecules, which results in a different ability to strip parts of their solvation shell and to form ion pairs. Ninham et al. [24] proposed to extend Collins rule with the consideration of dispersion interactions, while Vlachy et al. [19] showed a Hofmeister like ordering for charged headgroups and compared theoretical insights on the Gibbs free energy difference ΔΔG with Collins rule and showed that the tendency of the contact ion pair formation e.g., between sulfate groups and alkali ions increases with the size of the cation Li^+^ < Na^+^ < K^+^ < Rb^+^ < Cs^+^. In order to address the latter effects and their role at surfactant-modified air–water interfaces, sodium dodecyl sulfate (SDS) surfactants can be an important role model. Despite its possible contamination by dodecanol due to synthesis but also due to hydrolysis, SDS requires special care regarding purity [5], SDS is one of the most studied anionic surfactant and a topic of current research [25,26,27,28]. In the past, the structure of SDS monolayers at the air–water but also the oil–water interface was already investigated with the vibrational sum-frequency generation (SFG) [25,29,30,31,32,33], where both the interfacial water structure via O-H stretching vibrations [31] and the surfactant structure and coverage using C-H and S-O stretching vibrations were probed [25,34,35,36,37,38,39].

In our approach, we have studied the adsorption of DS^−^ surfactants to the air–water interface in the presence of different alkali ions using vibrational SFG spectroscopy. We show that the concentration dependence of both the frequency and SFG amplitude from S-O stretching vibrations can be used to quantify ion binding and surface coverage of the surfactant adlayer at the air–water interface.

## 2. Results and Discussion

**Figure 1:**
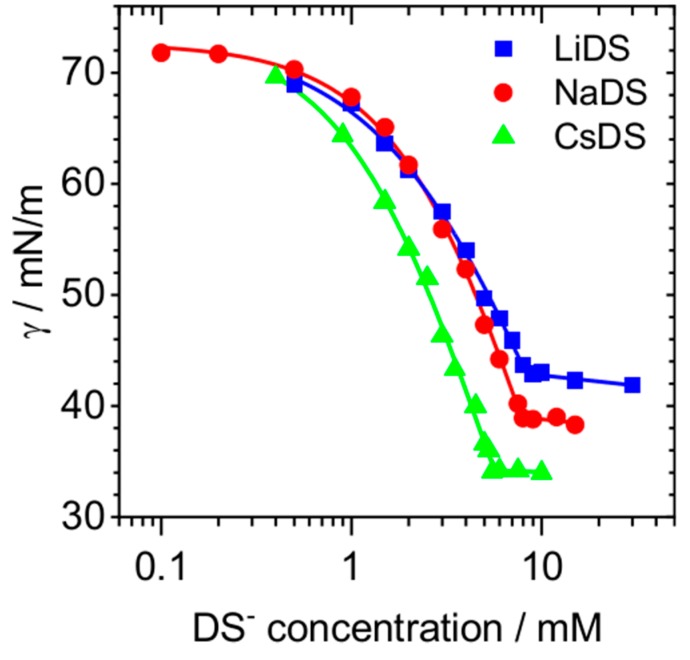
Surface tension isotherms at 295 K of dodecyl sulfate surfactants at the air–water interface with Li^+^, Na^+^ and Cs^+^ counter ions.

In Figure 1, surface tension isotherms of DS^−^ surfactants with Li^+^, Na^+^ and Cs^+^ counterions are presented. A close inspection of Figure 1 shows that the nature of the cation has a substantial effect on the surface tension of DS^−^ surfactants. From the analysis of Figure 1, several points can be noted: (i) The critical micelle concentration (CMC), which can be identified by the kink in the surface tension isotherm, is at 5.7 mM (CsDS), 8.1 mM (SDS) and 8.4 mM (LiDS). This is in excellent agreement with an earlier study by Lu et al. [40] who have reported CMCs of 8.7 mM (298 K), 8.2 mM (298 K), 6.7 mM (306 K), 5.9 mM (306 K) and 5.9 mM (306 K) for LiDS, SDS, KDS, RbDS and CsDS and is also confirmed by the surface tension isotherms as presented in the work by Schelero et al. [17] and by the report of Mysels et al. [8]. Clearly, the surfactant CMC increases in the order of Cs^+^ < Na^+^ < Li^+^ and (ii) is accompanied by an apparent decrease in the equilibrium constant K and Gibbs free energy for the adsorption of DS^−^ ions at the air–water interface in the same order as the CMC increases. We further point out, that all three isotherms in Figure 1 show a well-defined kink at the CMC. From that, we conclude that known effects through dodecanol impurities do not impair our results within the sensitivity limits of the surface tensiometry.

In order to investigate possible specific ion effects on the interface adsorption of dodecyl sulfate ions (DS^−^), we have performed SFG spectroscopy at the air–water interface that was modified with LiDS, SDS and CsDS in the presence and absence of the corresponding LiCl, NaCl, and CsCl salts. For that, we have concentrated on S-O stretching vibrations of the surfactants sulfate headgroup. Previously, Johnson and Tyrode [34] concluded from their detailed study of S-O stretching vibrations of SDS surfactants at the air–water interface, that orientation and ordering effects are for S-O stretching vibrations of SDS surfactants at the air–water interface negligible and independent of the SDS bulk concentration. In fact, their analysis of SFG spectra which were recorded with different polarization combinations did not indicate any orientation changes in the concentration range from 1 mM to concentrations well above the CMC of ~8.1 mM. 

For that reason, the amplitude of S-O stretching bands from dodecyl sulfate ions at the air–water interface is in this case only dependent on the surfactants surface excess. Addressing S-O stretching bands in the SFG spectra of DS^−^ ions at the air–water interface and in the presence of different cations and salts is therefore an ideal model system to investigate the role of specific ion effects on the interface adsorption of a simple ionic surfactant.

**Figure 2:**
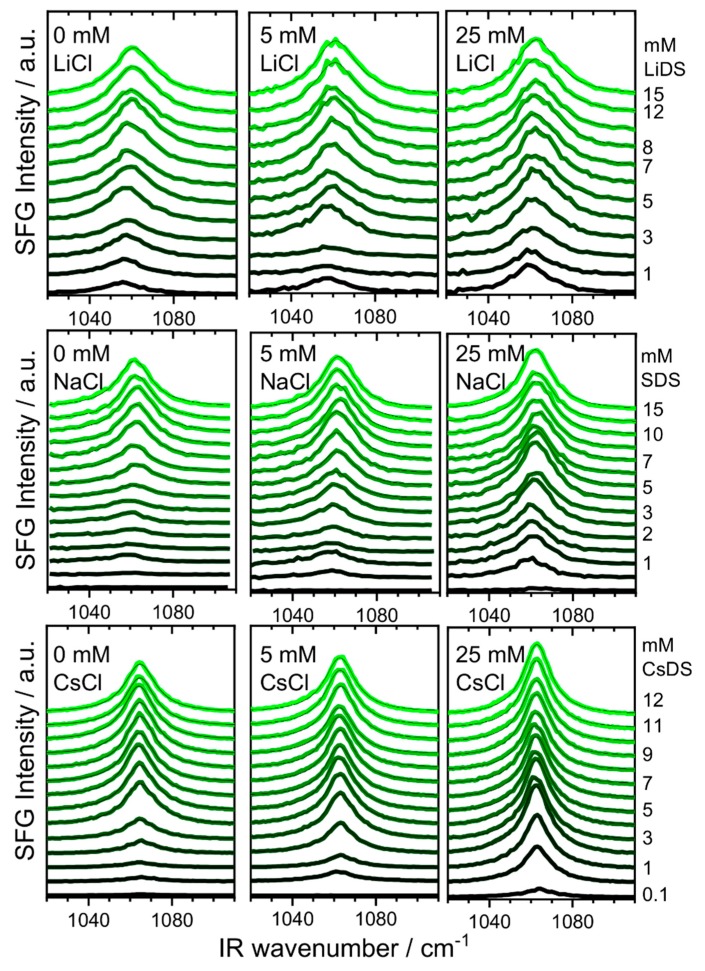
Vibrational sum-frequency generation (SFG) spectra of S-O stretching bands from dodecyl sulfate surfactants at the air–water interface in the presence of Li^+^, Na^+^ and Cs^+^ cations. For better visibility, a constant offset to the baseline was added. As indicated in the figure the surfactant concentration varied from very low concentrations to a concentration well above the critical micelle concentration (CMC), while 0 mM, 5 mM and 25 mM LiCl, NaCl and CsCl electrolytes were added to the lithium dodecyl sulfate (LiDS), sodium dodecyl sulfate (SDS) and cesium dodecyl sulfate (CsDS) solutions, respectively. The solid black lines correspond to the fitted SFG spectra using a Lorentzian line shape as explained in the text.

In Figure 2, we present the SFG spectra of the air–water interface as a function of LiDS, SDS and CsDS surfactant and LiCl, NaCl and CsCl salt concentrations. The SFG spectra in the presented frequency region are dominated by a single vibrational band that is centered between 1055 cm^−1^ and 1070 cm^−1^, has a typical full width at half maximum (FWHM) bandwidth of 20 cm^−1^ and is attributable to S-O stretching vibrations of the surfactants sulfate head group. In fact, even a visible inspection of Figure 2 reveals that the frequency of the S-O stretching band is a function of the LiDS and SDS surfactant concentration as well as the LiCl and NaCl electrolyte concentration. We will analyze the latter in more detail below.

From Figure 2, it can be also seen that the changes in the SFG spectra of the sulfate headgroup take place in a much narrower concentration range for the CsDS and SDS surfactants. The presence of an additional background electrolyte shifts the first rise of the S-O amplitude above the noise limit towards smaller concentrations. In fact, the latter is dominated by the more effective screening of the interfacial charges, which reduces the prevailing repulsive electrostatic interactions between the surfactant head groups at the air-water interface and thus favours surfactant adsorption at smaller concentrations. This is a well-known effect which also affects the surfactants CMC in the bulk solution. Previous works have demonstrated a decrease in CMC when an additional electrolyte is present. In the case of SDS surfactants, the native CMC at ~8 mM is decreased in the presence of an additional electrolyte to ~1 mM when >200 mM NaCl was added [41] Interestingly, there were no substantial differences when only the anionic species (chloride, acetate, or butyrate) was varied.

Already the results from surface tensiometry clearly demonstrate that the choice of the cationic species is important for the DS^−^ adsorption. This is consistent with Figure 2, where the changes in the SFG spectra between the LiDS, SDS and CsDS are compared. Here, the band positions and the onset for the DS^−^ adsorption at the air–water interface are different for the Li^+^, Na^+^ and Cs^+^ cations. In order to address the latter changes in more detail and on a more quantitative level, we have fitted the SFG spectra in Figure 2 using Lorentzian model functions according to Equations (7) and (8) (see Materials and Methods section). In our fitting procedures we have allowed the SFG amplitude $A_{q},$ the resonance frequency $\omega_{q},$ as well as the bandwidth $\gamma_{q}$ of the S-O stretching band as free parameters. 

**Figure 3:**
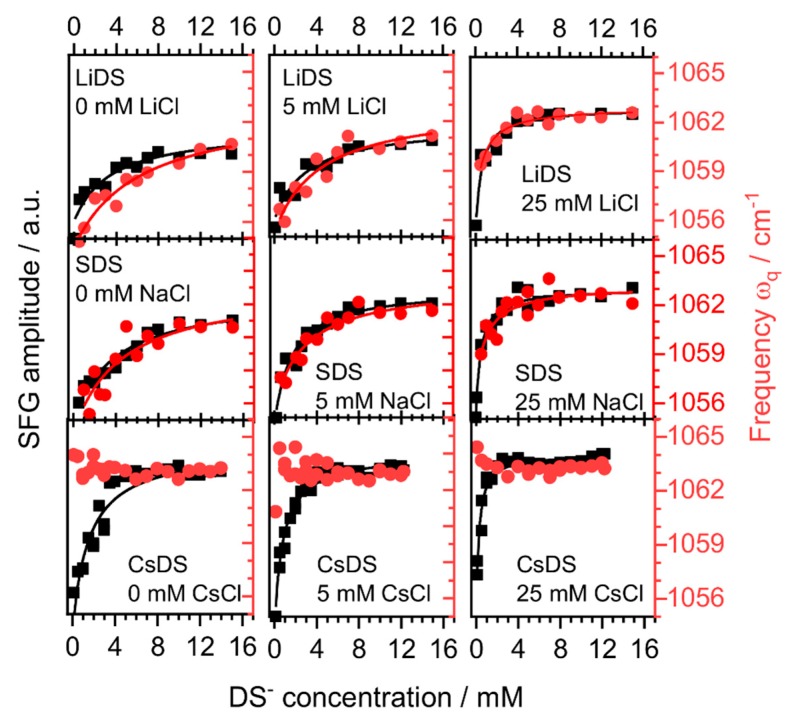
SFG amplitudes (black squares) and resonance frequencies (red circles) of LiDS, SDS and CsDS as a function of the surfactant and electrolyte (LiCl, NaCl and CsCl) concentration. The solid black (amplitude) and red (frequency) lines indicate the corresponding Langmuir fits.

In particular, the changes in $\gamma_{q}$ of the S-O band were negligible and a value of ~20 ± 4 cm^−1^ was consistently found. However, both the amplitude and resonance frequency of the S-O stretching band varied substantially, which is clearly shown by the summary of our fitting results in Figure 3.

First, we will now concentrate on the changes in SFG amplitudes which do display the expected behavior. In fact, Johnson and Tyrode who have already studied the SDS adsorption to the air–water interface using the SFG spectroscopy of the S-O stretching vibrations, have found similar changes as shown by our own results for SDS (Figure 3). However, we have now extended the experiments and their analysis to the presence of different cations. For a further discussion of the experimental results we recall that the SFG amplitude $A_{q}\propto \Gamma \langle \beta_{q}^{\left(2\right)}\rangle$ of a vibrational band is a function of the molecules surface excess Γ and the orientational average of the molecular hyper-polarizability $\langle \beta_{q}^{\left(2\right)}\rangle$ (for more information see the Material and Methods section). Since changes in $\langle \beta_{q}^{\left(2\right)}\rangle$ as a function of the concentration can be neglected, the amplitude $A_{q}$ is for DS^−^ ions at air–water interfaces dominated by the surface excess [34]. This enables us to perform a thermodynamic analysis of $A_{q}$ by applying a rather simple Langmuir adsorption isotherm (Equation (1)).

(1) θ=ΓΓ∞=KCCs1+KCCs

Here, *K* is the equilibrium constant for the adsorption at the air–water interface, θ the surface coverage, Γ the surface excess, $\Gamma_{\infty }$ is the limiting surface excess at the surfactant CMC, C the surfactant bulk concentration and $C_{s}$ the concentration of the aqueous solvent which is 55.5 M. Although other models like a Frumkin isotherm are more physically reasonable, we decided for a Langmuir model as it has only the equilibrium constant as a free parameter.

Fitting the changes in $A_{q}$ as a function of the concentration with the Langmuir isotherm (Equation (1)) shows excellent agreement with our experimental data. For each system the equilibrium constant and from the latter the apparent Gibbs free energy (ΔG=−RTln(K)) were determined (Table 1). We will now address the changes in the S-O stretching frequency $\omega_{q}$ which are shown in Figure 3. Note that the SFG amplitudes $A_{q}$ and $\omega_{q}$ are plotted in Figure 3 in a way, e.g., by multiplying with a constant factor so that there is a large overlap between the amplitude and frequency for many concentrations. A comparison of the concentration dependence of $A_{q}$ and $\omega_{q}$ reveals that this overlap is almost complete for the LiDS and SDS surfactants, whereas for CsDS the S-O stretching frequency did not change with the concentration and thus an overlap of the data was not possible. In all other cases, excellent agreement between the data sets were found. Consequently, compared to $A_{q}$ very similar equilibrium constants for the adsorption and Gibbs free energies were obtained from the analysis of $\omega_{q}$ using the Langmuir isotherm (justification below). Our analysis $\omega_{q}$ shows that a maximum shift in frequency of ~6 cm^−1^ can be observed and that at the highest DS^−^ concentrations and in the case of CsDS for all concentrations a limiting value of 1063 cm^−1^ was established.

We will now discuss a model that explains why the change in $\omega_{q}$ with the dodecyl sulfate concentration is dependent on the surfactants surface excess Γ. We relate the change in frequency to the vibrational Stark effect [42,43,44] of the surfactants sulfate head group within in the static electric field E of the interfacial electric double layer. In fact, sulfate anions are known to exhibit extraordinary strong electrochemical Stark tuning as shown for (bi)sulfate ions at the Pt(111)-electrolyte interface under potential control [45,46]. For that reason, it is likely that the changes in the interfacial electric field when the surfactants surface excess and thus the charging condition of the air–water interface increase, can also cause a noticeable shift in the S-O stretching frequency. 

In general, the frequency change $\Delta \omega_{q}$ within an external electric field E is given by

(2) $$\hbar \Delta \omega_{q}=-\Delta \vec{\mu }\cdot \vec{E}-\vec{E}\cdot \frac{\Delta \vec{\alpha }}{2}\vec{E}$$

where $\Delta \vec{\mu },$ $\Delta \vec{\alpha }$ and $\vec{E}$ are the changes in the dipole moment and polarizability between the ground and excited states as well as the external electric field [47]. As in many cases it has been shown that it is sufficient to approximate Equation (2) by the linear term in the external field, we can further simplify Equation (2) by just equating the linear term and reducing the equation to a one dimensional system perpendicular to the interface where the surfactant adsorption takes place:

(3) $$\hbar \Delta \omega_{q}=-\Delta \mu \cdot E$$

The external field is essentially given by the static electric field E inside the electric-double layer. In order to describe that field, we assume the Gouy-Chapman model of the electric double layer and a solution for the linearized Poisson-Boltzmann equation (Hückel approximation). In that case, we can write for the double-layer potential $\phi \left(z\right)=\phi_{0}e^{-\kappa z}$ with z being the coordinate perpendicular to the interface, $\phi_{0}$ the double-layer potential at z=0 and $\kappa =\lambda_{D}^{-1}$ the inverse Debye length. With that we can equate $\vec{E}=-\nabla \phi$ in our one-dimensional problem at z=0 in order to get the electric field $E_{0}=\kappa \phi_{0}$ directly at the interfacial plane (z=0). We are now using the Grahame equation for the charge density, which can be also linearized for low potentials ($\phi_{0}$ < 25 mV) in order to express the double layer potential by the charge density [48].

(4) $$\sigma =\sqrt{8C\varepsilon \varepsilon_{0}RT}\sinh\left(\frac{e\phi_{0}}{2k_{B}T}\right)\approx \frac{\varepsilon \varepsilon_{0}\phi_{0}}{\lambda_{D}}=\varepsilon \varepsilon_{0}\kappa \phi_{0}$$

Using the surface excess of dodecyl surfactants we can also write $-e\eta \Gamma \approx \varepsilon \varepsilon_{0}\kappa \phi_{0}$ and thus $E_{0}=\kappa \phi_{0}\approx -\frac{e\eta \Gamma }{\varepsilon \varepsilon_{0}},$ which we insert into Equation (3) and get as a result the following expression for the frequency shift of S-O stretching vibrations from the dodecyl sulfate moieties at the air–water interface:

(5) $$\Delta \omega_{q}=-\frac{\Delta \mu \cdot E}{\hbar }=-\frac{\Delta \mu \cdot \kappa \phi_{0}}{\hbar }\approx \frac{e\Delta \mu }{\varepsilon \varepsilon_{0}\hbar }\cdot \eta \cdot \Gamma$$

The above expression directly shows the linear relation between the surface excess Γ, the frequency shift $\Delta \omega_{q}$ as well as the degree of dissociation η between the DS^−^ surfactants and alkali cations. Clearly, the frequency shift in Figure 3 can be well described by Equation (5), since there is also an excellent overlap between the changes in amplitude and frequency for LiDS and SDS, which brings strong support to our model. For that, we recall that the SFG amplitude is also linearly dependent on Γ for the system under investigation [34]. In contrast to the Li^+^ and Na^+^ cations, the presence of the Cs^+^ ions did not cause any visible shifts in the S-O stretching frequency of the interfacial dodecyl sulfate ions. However, in the case of Cs^+^, the S-O stretching frequency was identical to the limiting frequency at concentrations near or above the respective CMCs of the LiDS and SDS. Apparently for the CsDS surfactants, the local electric field at the surfactant sulfate head group must be already at low concentrations similar to that of the interfacial LiDS and SDS at concentrations where the limiting surface excess and thus charge density is established at the interface. We associate this local field in the case of Cs^+^ to the increased concentration of cations in the electric double layer due to the ion pair formation. Clearly, for the CsDS ion pairs or solvent-shared ion pairs are formed and modify the interfacial electric field substantially. As a consequence, the degree of dissociation η must drop to a minimal value. However, we must also point out that the above described model breaks down as such ion specific effects like the ion pair formation cannot be explained by the applied Gouy-Chapman model. We take the absence of any frequency shift with the surfactant concentration and the fact that already at low concentrations the same frequency is found for CsDS as for LiDS and SDS at very high concentrations as clear evidence for the ion pair formation. The tendency to form ion pairs with dodecyl sulfate ions is for Cs^+^ higher compared to Li^+^ and Na^+^ and is well in line with the expectation from the Collins rule of matching water affinities [22,24,49]. Similar ordering of interactions between alkali cations with uncharged carboxylic acid groups from fatty acid monolayers at the air–water interface is reported in the work by Sthoer et al. [6], which also show that this ordering of ion interactions is reversed when the layer is charged with a high density of carboxylate groups.

**Figure 4:**
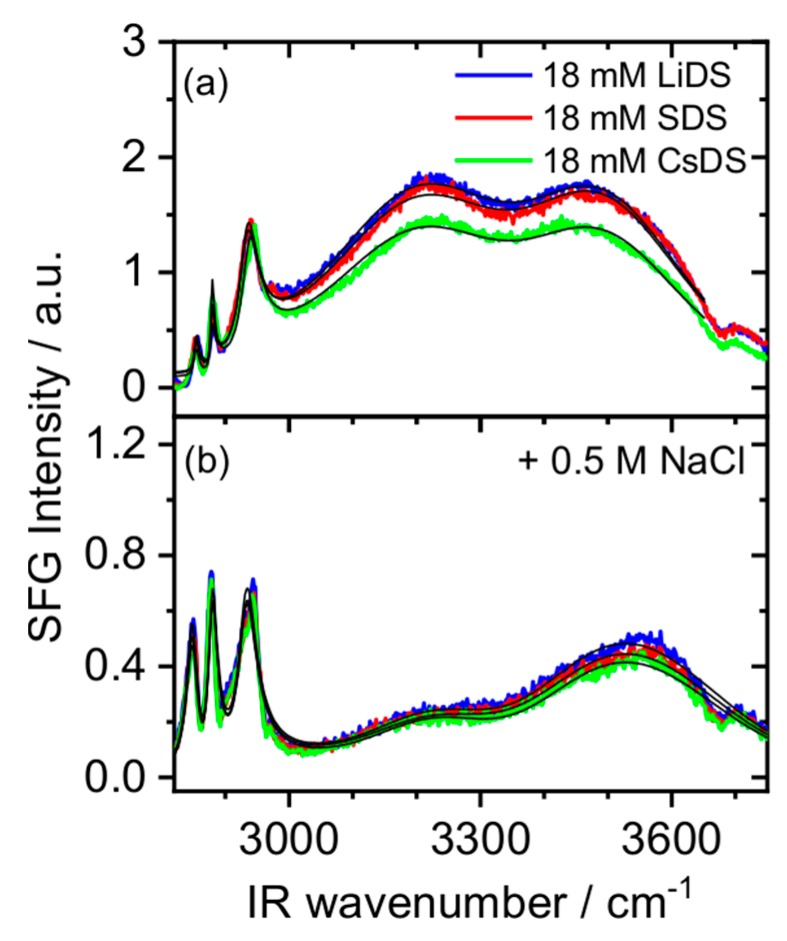
Vibrational SFG spectra of air-water interfaces with 18 mM LiDS, SDS and CsDS (**a**) without additions of salt and (**b**) in presence of 0.5 M NaCl. The black solid lines represent the corresponding fits to the SFG spectra according to Equations (7) and (8).

In order to probe the charging state at the electric double layer, we have performed additional SFG experiments in the frequency region of C-H and O-H stretching bands (2800 cm^−1^–3700 cm^−1^). In Figure 4a, the SFG spectra of air–water interfaces with 18 mM LiDS, SDS and CsDS are shown. The vibrational bands at 2854 cm^−1^, 2881 cm^−1^ and 2940 cm^−1^ can be assigned to the CH_2_ (ss), CH_3_ (ss) and CH_3_ (F) of DS^−^, respectively. Two broad bands centered around 3200 cm^−1^ and 3470 cm^−1^ can be assigned to the O-H stretching vibrations of interfacial water molecules [31] Here, O-H intensities provide information about the charging state in the electrical double layer [50,51,52]. Increasing the interfacial charge, e.g., by increasing the surface excess of the dissociated DS^−^ will cause an increase in the static electric field within the electric double layer, and can also cause additional polar ordering of interfacial water molecules. As a result, the O-H intensity becomes dependent on the interfacial electric field and several previous works have actually exploited the latter in order to get qualitative or in some cases even quantitative information on the double layer charging from the SFG spectra [6,51,52]. For more details on the principles of this method the reader is referred to the Materials and Methods section. From Figure 4a it can be clearly seen, that the latter decreases in the order Li^+^ > Na^+^ > Cs^+^.

By separating the contributions of the second- and third-order electric susceptibility $\chi_{S}^{\left(2\right)}$ and $\chi_{S}^{\left(3\right)}$ in Equation (7) (see below) we can extract a value of the double layer potential $\phi_{0}$. This procedure is described in detail elsewhere [52] but will explained briefly below. 

In order to obtain the $\chi_{S}^{\left(2\right)}$ contribution, we have performed SFG experiments at a similar surfactant and high salt concentration of 0.5 M NaCl (Figure 4b). Here, the static electrical field is screened by the addition of salt so that the electric field induced contribution vanishes. In this case, we assume that the O-H amplitude of Figure 4b becomes proportional to the contribution of $\chi_{S}^{\left(2\right)}$. At low ionic strength (Figure 4a), the O-H amplitudes are governed by the effective susceptibility $\chi_{eff}^{\left(2\right)}$ contribution of $\chi_{S}^{\left(2\right)}$ and $\chi_{S,EDL}^{\left(2\right)}$. Since the double layer potential of SDS above the CMC is already known in the literature [53,54] ($\phi_{0}$ = 82 mV), we can extract a value of the $\chi^{\left(3\right)}$ contribution by the O-H amplitudes in the SFG spectra. After this ‘calibration’, we know $\chi_{S}^{\left(2\right)}$ and $\chi_{S}^{\left(3\right)}$ and can determine the apparent double layer potentials for LiDS (83 mV) and CsDS (75 mV) surfactants from the SFG amplitude.

If we now calculate the surface charge density σ by the help of Grahames Equation (4), we can estimate the degree of dissociation η for the DS^−^ surfactants and alkali ions using the surface excess Γ as shown in Table 1:

(6) $$\eta =\frac{\sigma }{e\cdot \Gamma }$$

For the different counterions we found a degree of dissociation of 12.0% (LiDS), 10.4% (SDS) and 7.7% (CsDS) which is well in line with Collins ‘rule of matching water affinities’ and our above-mentioned discussion. However, the difference in η for the Li^+^, Na^+^ and Cs^+^ ions, which comes from an analysis of the O-H stretching vibrations and is associated to the SFG signal of water molecules within the interfacial double layer is substantially different from the observed Stark shift. The latter shift is maximized for Cs^+^ at even the lowest concentration and consequently no further changes in the frequency are observed when the Cs^+^ concentration increases. In terms of frequency shifts, the effect of Li^+^ and Na^+^ is similar but much less pronounced as compared to Cs^+^. By addressing the Stark shift, the local electric field of the surfactant headgroup is probed and we can thus gain direct information on the ion pair formation. In contrast, probing the mean electric field within the electric double layer by analyzing the O-H stretching vibrations contains indirect information on the surfactants’ charging state. As a result of the latter, we can only gain information on an apparent double layer potential that corresponds to the reduced charge at the interface due to the ion pair formation.

## 3. Materials and Methods

### 3.1. Sample Preparation

Sodium dodecyl sulfate (SDS, >99%) and lithium dodecyl sulfate (LiDS, >99%) surfactants were purchased from Fisher Scientific (Pittsburgh, PA, USA) and Carl Roth (Karlsruhe, Germany), respectively. SDS was recrystallized three times in water and ethanol before usage. Cesium dodecyl sulfate (CsDS) was synthesized using the method described by Schelero et al. [17]. The purity of surfactants was checked by recording surface tension isotherms (Figure 1). NaCl (>99.5%, Sigma Aldrich, St. Louis, MS, USA), LiCl (>99.2%, VWR, Radnor, PA, USA) and CsCl (>99.999%, Carl Roth, Karlsruhe, Germany) salts were used as received. 

Stock solutions were prepared by dissolving the necessary amount of surfactants and salts in ultrapure water (18 MΩ∙cm; total oxidizable carbon <5 ppb), which was obtained from a Milli-Q Reference A+ (Merck, Darmstadt, Germany) purification system. Subsequently, the stock solutions were sonicated until dissolution was reached. The required glassware was cleaned with the Alconox detergent solution (Sigma Aldrich), dried and afterwards stored in concentrated sulfuric acid (98% p.a., Carl Roth) with NOCHROMIX (Godax Labs, Bethesda, MD, USA) for at least 12 h. The acid-cleaned glassware was rinsed with copious amounts of ultrapure water and subsequently dried in a stream of 99.999% N_2_ gas (Westfalen Gas, Münster, Germany).

### 3.2. Sum Frequency Generation (SFG)

SFG is a powerful tool in order to probe surfaces and interfaces at the molecular level. By overlapping a tunable broadband (>300 cm^−1^ full width at half maximum (FWHM)) femtosecond infrared (IR) pulse with the frequency $\omega_{IR}$ and a narrowband (4 cm^−1^, FWHM) visible picosecond pulse (VIS) with the frequency $\omega_{VIS}$ spatially and temporally at the sample, a third beam with the sum frequency $\omega_{SF}=\omega_{IR}+\omega_{VIS}$ is generated. The sum frequency intensity $I_{SF}$ is a function of the intensities of the incoming beams and the non-resonant $\chi_{NR}^{\left(2\right)}$ and effective part $\chi_{eff}^{\left(2\right)}$ of the second-order susceptibility [50,51,52].

(7) $$I_{SF}\propto {\left|\chi_{NR}^{\left(2\right)}+\chi_{eff}^{\left(2\right)}\right|}^{2}={\left|\chi_{NR}^{\left(2\right)}+\chi_{S}^{\left(2\right)}+\frac{\kappa }{\kappa +i\Delta \kappa_{z}}\chi_{S}^{\left(3\right)}\phi_{0}\right|}^{2}$$

(8) $$\;\chi_{S}^{\left(2\right)}=\underset{q}{\sum }\frac{A_{q}}{\omega_{q}-\omega_{IR}+i\gamma_{q}}$$

$\;\chi_{eff}^{\left(2\right)}$ is equal to $\chi_{S}^{\left(2\right)}+\chi_{S}^{\left(3\right)}\phi_{0}$ if the ionic strength is >1 mM (here the prefactor $\frac{\kappa }{\kappa +i\Delta \kappa_{z}}$ in Equation (7) is equal to 1) and is given by the second-order susceptibility $\chi_{S}^{\left(2\right)},$ which is dominated by the molecular structure of the interfacial molecules and a contribution of the electrical double layer $\chi_{S}^{\left(3\right)}$. The spectral line shape of $\chi_{eff}^{\left(2\right)}$ can be expressed by a coherent overlap of bands with Lorentzian line shapes (in the case of the O-H stretching bands Voigt profiles in order to account for inhomogeneous broadening) and is a function of the resonance frequency $\omega_{q}$, the Lorentzian line width $\gamma_{q}$ and of the amplitude $A_{q}$ of the q-th vibrational mode. The oscillator strength $A_{q}\propto \Gamma \langle \beta_{q}^{\left(2\right)}\rangle$ depends on the molecules’ hyper polarizability $\beta_{q}^{\left(2\right)}$, its orientational average $\langle \beta_{q}^{\left(2\right)}\rangle$ and the surface excess Γ of interfacial molecules. In the isotropic bulk solution, the orientational average of $\beta_{q}^{\left(2\right)}$ is zero and consequently the SF signal vanishes. Since interfacial molecules at the interface are preferentially orientated, the orientational average of $\langle \beta^{\left(2\right)}\rangle$ from interfacial molecules is nonzero and, thus, SFG is inherently interface specific for the isotropic and centrosymmetric materials. The adsorbed charged molecules cause an electrostatic field, which points perpendicular to the surface and leads to a reorientation and polarization of water molecules in the diffuse double layer. This contribution to the SFG intensity is described in Equation (7) by the inverse Debye length κ, the wave vector mismatch $\Delta \kappa_{z}$, the third order susceptibility $\chi_{S}^{\left(3\right)}$ and the double layer potential $\phi_{0}$.

In this work, the SFG experiments were performed with a home-built device that is described in detail elsewhere [52]. In brief, the spectrometer consists of the Spectra Physics (SolsticeAce) amplifier system that is seeded with a Spectra Physics (MaiTaiSP) femtosecond oscillator and generates 70 fs pulses at a repetition rate of 1 kHz. The pulse energy of the amplified beam is split and 3.5 mJ are used to pump an optical parametric amplifier (TOPAS Prime, Light Conversion) with a subsequent non-collinear difference frequency generation for signal and idler photons from the TOPAS. This yields tunable mid-IR pulses. For the experiments in the vibrational S-O region, the >300 cm^−1^ broadband mid-IR pulse was centered at a frequency of 1050 cm^−1^ and had a pulse energy of 8 µJ. the SFG spectra in the spectral range between 2800 cm^−1^ to 3700 cm^−1^ was recorded by scanning five IR frequencies. The 4 cm^−1^ narrowband ‘visible’ pulse at a wavelength of 804.1 nm and with a pulse energy of 20 µJ was generated from the remainder of the pulse energy coming from the amplifier systems by spectral filtering the beam with an etalon. The IR and visible beams were overlapped and focused to the air–water interface at 60° and 55° angles of incidence, respectively. The reflected sum-frequency beam was collected and guided to a spectrograph (Andor Kymera), where it gets spectrally dispersed with a 1200 lines/mm grating and is subsequently recorded with an EMCCD (Andor, Newton). The total acquisition time for each sample was <2 min but varied with the surfactant concentration. All spectra were recorded at 295 K room temperature by placing 3 mL sample solution in a Petri dish with a diameter of 30 mm. A ssp polarization combination with the s-polarized sum frequency, s-polarized visible and p-polarized IR beams was used, and the SFG spectra were referenced to the non-resonant SFG signal of an air-plasma cleaned gold film on top of a Si wafer. In order to record the Au reference spectrum, ppp polarizations were used.

### 3.3. Surface Tensiometry

The surface tension of the air–water interface at 295 K was determined with the pendant drop method using a PAT1M device (Sinterface, Berlin, Germany) by analyzing the drop shape with an application of the Young–Laplace equation. In order to minimize possible effects of incomplete adsorption kinetics, the surface tension was continuously recorded as a function of time and reported after an adsorption time of 10,000 s.

## 4. Conclusions

The adsorption of dodecyl sulfate surfactants at the air–water interface in the presence of the Li^+^, Na^+^ and Cs^+^ cations was studied with a vibrational SFG and surface tensiometry. The SFG amplitude and the resonance frequency of the S-O stretching vibration was used in order to trace the changes in surfactant’ surface excess at the interface. LiDS and SDS showed an excellent agreement in the changes of the SFG amplitude and S-O resonance frequency with a surfactant concentration. The frequency shift is due to a Stark shift that the interfacial surfactants experience within the interfacial electric double layer. Using a Langmuir adsorption model, we have calculated the Gibbs free energy of the adsorption and found no substantial differences between the LiDS and SDS surfactants, while the Cs^+^ cations showed a nearly constant resonance frequency for different surfactant concentrations. We explain the latter with the formation of ion pairs or solvent-shared ion pairs between the Cs^+^ cations and the sulfonate headgroups. This reduces the degree of dissociation of the DS^−^ surfactants and Cs^+^ cations and minimizes the electrostatic repulsive forces in the double layer, which favours a higher surface excess and Gibbs free energy. The change in the double layer potential for the different counterions was shown by analyzing the SFG signal of the O-H vibrations. By applying Grahame’s equation, we were able to calculate the surface charge density and in combination with the known surface excess the specific degree of dissociation. Here, we found 12.0%, 10.4% and 7.7% for LiDS, SDS and CsDS, respectively. The fact that the possibility of the ion pair formation is higher for Cs^+^ than for Na^+^ and Li^+^ is in well agreement with the proposed ‘rule of matching water affinities’ by Collins.

## Figures and Tables

**Table 1:** CMC (tensiometry and SFG), surface excess (from reference [40]) and averaged (frequency and amplitude) as well as Gibbs free energies calculated from the analysis of the results in Figure 3.

	ΔG\(kJ/mol)	CMC\mM	$\;\Gamma_{\infty }\text{\textbackslash{}}(µM\text{\textbackslash{}}m²)$
LiDS	−23.4	8.4	3.32
LiDS + 25 mM LiCl	−28.2	5.3	
SDS	−23.6	8.1	3.77
SDS + 25 mM NaCl	−28.2	3.7	
CsDS	−26.4	5.1	4.37
CsDS + 25 mM CsCl	−30.7	2.3	
